# *Staphylococcus aureus* vs. Osteoblast: Relationship and Consequences in Osteomyelitis

**DOI:** 10.3389/fcimb.2015.00085

**Published:** 2015-11-26

**Authors:** Jérôme Josse, Frédéric Velard, Sophie C. Gangloff

**Affiliations:** EA 4691 Biomatériaux et inflammation en site osseux, Pôle Santé, Université de Reims Champagne-ArdenneReims, France

**Keywords:** *Staphylococcus aureus*, osteoblast, internalization, inflammation, antibiotics, antimicrobial peptide, phenol soluble modulin, small colony variants

## Abstract

Bone cells, namely osteoblasts and osteoclasts work in concert and are responsible for bone extracellular matrix formation and resorption. This homeostasis is, in part, altered during infections by *Staphylococcus aureus* through the induction of various responses from the osteoblasts. This includes the over-production of chemokines, cytokines and growth factors, thus suggesting a role for these cells in both innate and adaptive immunity. *S. aureus* decreases the activity and viability of osteoblasts, by induction of apoptosis-dependent and independent mechanisms. The tight relationship between osteoclasts and osteoblasts is also modulated by *S. aureus* infection. The present review provides a survey of the relevant literature discussing the important aspects of *S. aureus* and osteoblast interaction as well as the ability for antimicrobial peptides to kill intra-osteoblastic *S. aureus*, hence emphasizing the necessity for new anti-infectious therapeutics.

## Introduction

Bone is quantitatively the most abundant mineralized tissue in humans. It is composed of cells evolving in a tangle of collagen and non-collagenous proteins, which can be mineralized to form the bone extracellular matrix (BEM). Osteoblasts are the bone forming cells. First, osteoblasts synthesize an organic matrix around themselves and mineralize it to create the mature BEM (Ecarot-Charrier et al., 1983) where they are ultimately integrated as osteocytes (Dallas and Bonewald, 2010). The synthesis of the BEM is balanced by a resorption performed by osteoclasts. Rising from the fusion of monocytic precursors to form giant multinuclear cells, osteoclasts secrete H^+^ protons and several proteases to resorb both inorganic and organic parts of the BEM (Edwards and Mundy, 2011). The concomitant bone formation and resorption create perpetual physiological homeostasis.

Osteoblast activity is altered in pathological conditions such as cancers, autoimmune diseases and infections. Osteomyelitis are damaging bone infections, that can lead to sequelae and if uncontrolled, to patients death. During childhood, osteomyelitis are mostly a result of hematogenous spread from distant infected tissues. In adults, surgical procedures are becoming increasingly responsible for a direct contamination of bone tissue, especially in presence of medical devices (Montanaro et al., 2011). The incidence of osteomyelitis varies from 1 to 55% in open fracture cases as a function of the grade of the fracture (Kim and Leopold, 2012; Hogan et al., 2013). Germ concentration, pathogenicity of the implicated pathogen or systemic factors such as nicotine, obesity or diabetes mellitus can also favor the prognosis of direct osteomyelitis (Hogan et al., 2013). Osteomyelitis are typically of bacterial origin, notably *Staphylococcus* species, where *Staphylococcus aureus* is the most prevalent pathogen (Lew and Waldvogel, 2004), also responsible for various community or hospital-acquired infections like skin abscess, pneumonia or septic arthritis (Lowy, 1998).

Infection of osteoblasts by *S. aureus* is a keystone, a determining element in the development of osteomyelitis in bone tissue and as such this review focuses on the interaction between *S. aureus* and the osteoblasts that play an important role in bone homeostasis. *S. aureus* directly interacts with osteoblasts both in the extracellular and intracellular space following its internalization (Hudson et al., 1995). Each interaction specifically leads to the induction of various and distinct responses from the osteoblasts.

*S. aureus* possesses on its surface an arsenal of pathogen-associated molecular patterns (PAMP) that interact with osteoblasts to induce the production of chemokines and cytokines that then recruit and activate innate and adaptive immune cells (Heilmann, 2011; Claro et al., 2013). In addition to inflammatory response, *S. aureus* can also induce the death of osteoblasts and increase osteoclastogenesis through osteoblasts stimulation thus resulting in an imbalanced bone homeostasis (Tucker et al., 2000; Widaa et al., 2012). The combination of these effects leads to inflammatory bone loss.

Recent studies have consequently focused on antibiotics and their ability to kill intracellular *S. aureus* as well as on antimicrobial peptides (AMP) that can be produced by osteoblasts (Varoga et al., 2009; Zhu et al., 2014) or chemically synthetized (Choe et al., 2015).

*S. aureus* embodies a major clinical challenge, as chronic osteomyelitis with *S. aureus* are difficult to treat with antibiotics (Spellberg and Lipsky, 2012). The ability of *S. aureus* to survive in osteoblasts after internalization can protect it from immune cells but also from the antibiotics that may not penetrate the cells (Valour et al., 2015a).

We hereby provide an overview of the relationship and interaction between *S. aureus* and osteoblasts, from the onset of the infection to new therapeutics.

## Bone extracellular matrix, a niche for *S. aureus*

**Figure 1:**
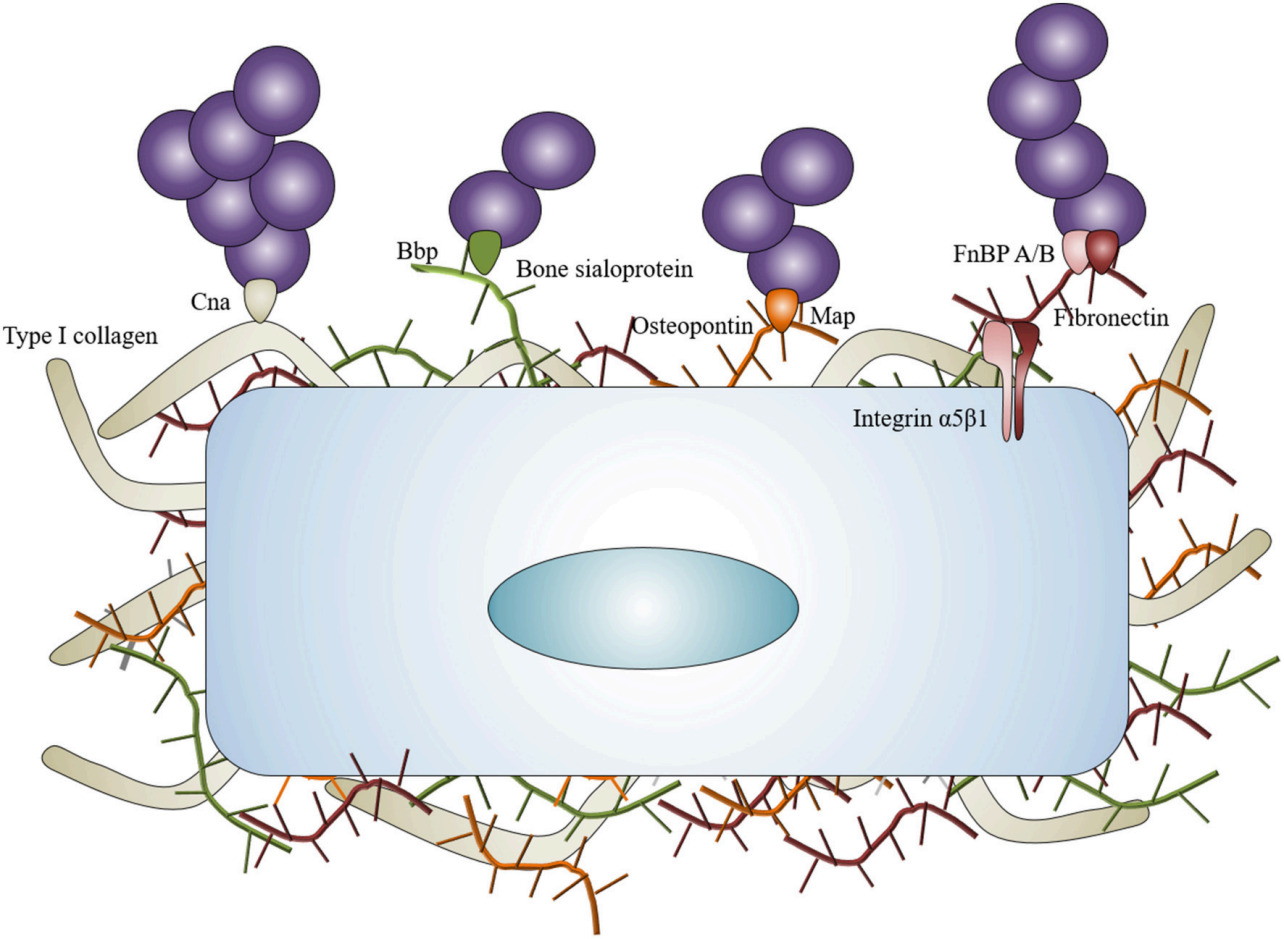
***Staphylococcus aureus* interaction with BEM**. *Staphylococcus aureus* can interact with the BEM to concentrate around osteoblasts. Collagen adhesin (Cna) links with type I collagen, bone sialoprotein binding protein (Bbp) links with bone sialoprotein, MHC II analog protein (Map) can potentially link osteopontin. Fibronectin binding proteins A and B (FnBP A/B) link with fibronectin and act as bridges between *Staphylococcus aureus* and osteoblasts through α5β1 integrin.

The capacity of *S. aureus* to infect bone—and more specifically the osteoblast—is tightly correlated to its ability to bind the BEM components, notably *via* multiple adhesins called the MSCRAMMs which stands for microbial surface components recognizing adhesive matrix molecules (Heilmann, 2011). The proteins and glycans present in the BEM are all potential *S. aureus* binding sites. The most studied of these are type I collagen, bone sialoprotein, osteopontin, and fibronectin because they directly interact with *S. aureus* (Figure 1).

Type I collagen represents approximately 90–95% of the organic fraction of the BEM, thus supporting a major role for the collagen adhesin (Cna) of *S. aureus* in the pathogenesis of osteomyelitis. Cna has resulted in being pivotal for settling osteomyelitis through hematogenous spread in a murine model (Elasri et al., 2002). Cna is highly expressed during the stage of exponential growth of *S. aureus* (Gillaspy et al., 1997) and Cna-positive strains are often associated with a slime formation, a state that enhances the pathogenicity of *S. aureus* in implant-associated osteomyelitis (Montanaro et al., 1999).

The bone sialoprotein represents however another means for *S. aureus* to bind the BEM. This protein constitutes approximately 8% of all non-collagenous proteins found in bone (Fisher et al., 1990). The bone sialoprotein binding protein (bbp) is a member of the staphylococcal serine-aspartate dipeptide repeat (sdr) family (Tung et al., 2000) and is one of the first adhesins studied for its potential role in osteomyelitis caused by *S. aureus* (Rydén et al., 1989). Bbp could be considered as an adhesin with several ligands due to the recognition of fibrinogen in a non-osseous context (Vazquez et al., 2011). The presence of both *cna* and *bbp* genes in *S. aureus* is considered as a major marker of virulence in isolates from implant-associated osteomyelitis (Campoccia et al., 2009).

An involvement of *S. aureus* Map—a functional bacterial analog to mammalian MHC class II molecules—in osteomyelitis is also speculated in regard to its ability to bind recombinant osteopontin (Jönsson et al., 1995). The ability of *S. aureus* to bind to biological osteopontin has nevertheless not been reported yet.

Fibronectin is the most studied component of the extracellular matrix in staphylococcal infection, especially in osteomyelitis. Fibronectin acts as a bridge between *S. aureus* and the osteoblasts. On the one hand, *S. aureus* holds two types of proteins that can bind to fibronectin on its surface: the fibronectin binding proteins A and B (FnBP A/B). On the other hand, fibronectin connects to the osteoblasts through the α5β1 integrin. This “fibronectin bridge” allows the attachment of *S. aureus* and also favors its internalization by non-phagocytic cells like many cell types (Dziewanowska et al., 1999; Peacock et al., 1999; Ahmed et al., 2001; Kintarak et al., 2004).

BEM attachment represents a major step in osteomyelitis onset as 10–60% of studied *S. aureus* clones express *cna, bbp*, and *fnbB* genes and 100% express *fnbA* or *clumping factor A and B* genes (Otsuka et al., 2006; Campoccia et al., 2009; Atshan et al., 2012; Post et al., 2014). In specific osteomyelitis case, *cna* and *bbp* genes were more frequently identified in non-implant related infections than in implant-related infections (Post et al., 2014). Of note, expression of *cna* gene is increased in methicillin resistant clones compared with methicillin sensible ones (Atshan et al., 2012). To illustrate this point, the combination of *cna* and *bbp* genes characterizes ST30—a pandemic community-acquired methicillin resistant *S. aureus* that causes osteomyelitis—(Otsuka et al., 2006; Dhanoa et al., 2012).

BEM is therefore an important actor in the first step of *S. aureus* infection as it is produced by and connected to osteoblasts. It allows the bacteria to bind and concentrate around osteoblasts, particularly for staphylococcal clones possessing *cna, bbp*, and/or *fnbB* genes.

## Invasion/internalization of *S. aureus* into osteoblasts

Ogawa et al. were the first to report the capacity of endothelial cells to “engulf” *S. aureus* (Ogawa et al., 1985). It has been shown since this observation that *S. aureus* can adhere to and invade cultured osteoblasts (Table 1), as well as osteoblasts and osteocytes *in vivo* (Hudson et al., 1995; Reilly et al., 2000; Bosse et al., 2005).

**Table 1:** ***Staphylococcus aureus* internalization by cultured osteoblasts**.

**Cells**	**Strains**	**MOI**	**Observations**	**Author/Year**
CECOs	Lab	-	Adherence, internalization and intracellular survival of *Staphylococcus aureus* inside osteoblasts	Hudson et al., 1995
MC3T3-E1	UAMS-1	-	Involvement of actin microfilaments, microtubules and clathrincoated pits in the internalization of *Staphylococcus aureus* by osteoblasts	Ellington et al., 1999
MG-63, NHOs	Lab, Clinic	30:1	Role of cytoskeleton of osteoblasts, notably actin microfilaments, in internalization of *Staphylococcus aureus*	Jevon et al., 1999
MG-63	Lab	300:1	Role of fibronectin binding proteins A/B (FnBP A/B) in the internalization of *Staphylococcus aureus* in osteoblasts	Ahmed et al., 2001
NHOs	UAMS-1	25:1, 75:1, 250:1	Release of intracellular *Staphylococcus aureus* by dead osteoblasts and ability to re-infect new osteoblasts	Ellington et al., 2003
MG-63	Lab	300:1	Correlation between SigB expression in *Staphylococcus aureus* and its capacity for internalization inside osteoblasts	Nair et al., 2003
MG-63	Lab	50:1, 200:1, 500:1	Internalization of *Staphylococcus aureus* into osteoblasts through a fibronectin bridge to integrin α5β1	Khalil et al., 2007
MG-63	Clinic	-	Synergistic role of Cna and Bbp adhesins in the initial adhesion of *Staphylococcus aureus* to osteoblasts, favoring the subsequent FnBPA-mediated internalization.	Testoni et al., 2011
pHOB	Lab, Clinic	50:1	*Staphylococcus aureus* can persist within osteoblasts for several weeks. Persistence induces bacterial phenotypic diversity, including SCV phenotypes, accompanied by changes in virulence factor expression	Tuchscherr et al., 2011
MG-63	Lab	10:1	Evidence of a trafficking of intracellular *Staphylococcus aureus* into the late endosomal/lysosomal compartment in osteoblasts	Jauregui et al., 2013
UMR-106	Lab	100:1, 500:1, 1000:1	Internalization and survival of *Staphylococcus aureus* within osteoblasts and macrophages led to differential responses	Hamza and Li, 2014
pHOB	Lab, Clinic	50:1	Chronic osteomyelitis isolates were characterized by a high host cell invasion rate, low cytotoxicity and the ability to persist and adapt within osteoblasts through SCVs formation	Kalinka et al., 2014
pHOB	Lab, Clinic	50:1	Sigma Factor SigB is an essential factor that enables *Staphylococcus aureus* to switch from the highly aggressive phenotype that settles an acute infection to a silent SCV-phenotype that allows for long-term intracellular persistence	Tuchscherr et al., 2015
pHOB	Lab, Clinic	50:1	*Staphylococcus aureus* continuously upregulated the expression of SigB during intracellular persistence. The increased SigB expression was accompanied by upregulation of adhesins and downregulation of toxins, which are characteristic for SCVs	Tuchscherr and Löffler, 2015
MG-63	Clinic	100:1	Lack of delta-toxin production was significantly associated with osteomyelitis chronicity, osteoblast invasion and biofilm formation	Valour et al., 2015b

MOI, Multiplicity of infection (number of bacteria per cell); CECOs, Chick embryo calvarial osteoblasts; NHOs, Normal human osteoblasts; pHOB, Primary culture of human osteoblasts; MC3T3-E1, Mouse calvaria cell line; MG-63, Human osteosarcoma cell line; UMR-106, Rat osteosarcoma cell line; Lab, Laboratory strains of Staphylococcus aureus (purchased from ATCC, NCTC,… ); Clinic, Strain of Staphylococcus aureus isolated from osteomyelitic patients; UAMS-1, Most studied laboratory strain of Staphylococcus aureus in bone infection model.

**Figure 2:**
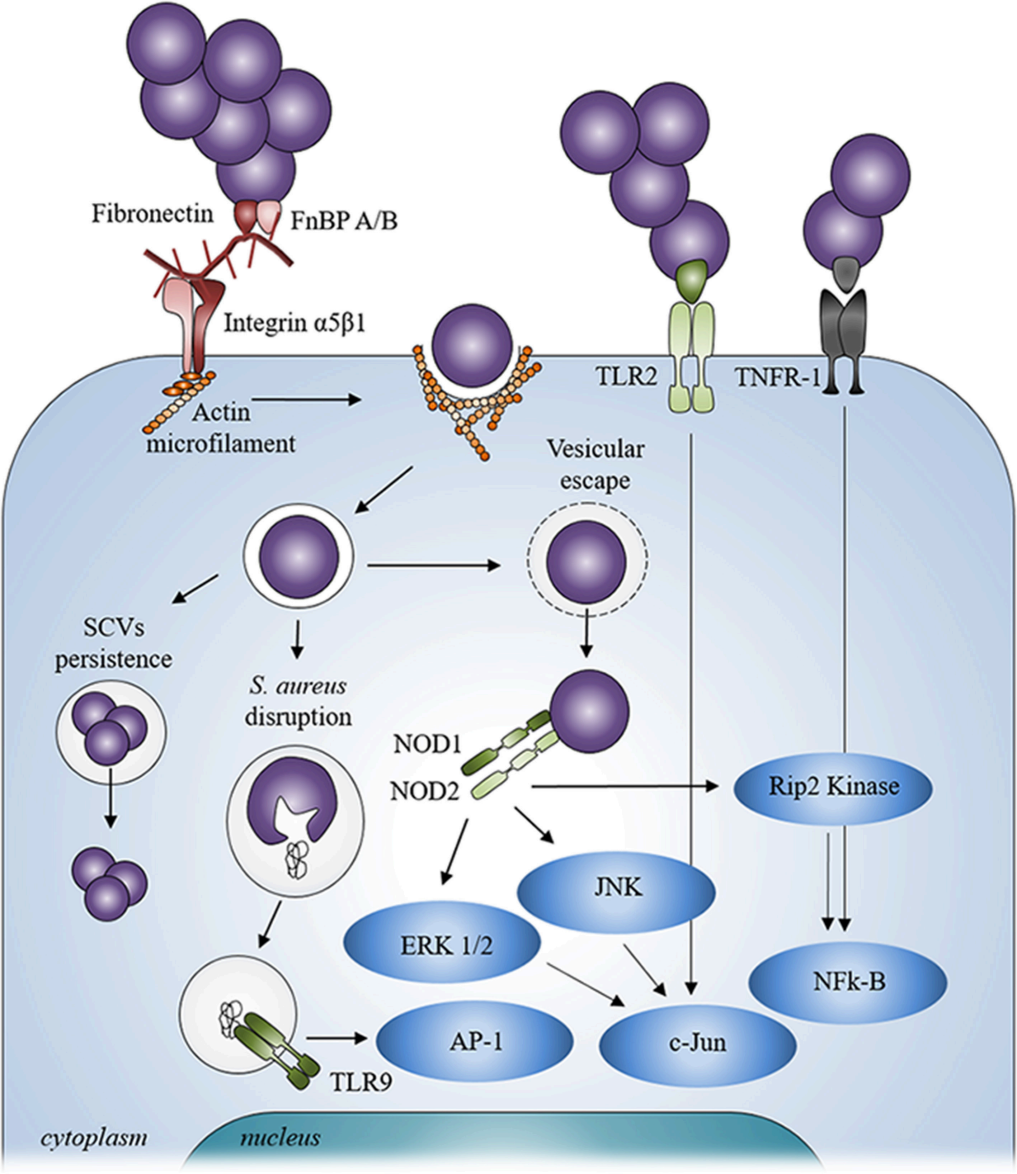
***Staphylococcus aureus* signaling mechanism after contact and internalization by osteoblasts**. After internalization, *Staphylococcus aureus* can escape from vesicle, be disrupted from inside the vesicle or persist inside osteoblasts through a SCV phenotype. *Staphylococcus aureus* can also interact with extracellular receptors TLR2 and TNFR-1 and with intracellular receptors TLR9 and NODs after its internalization into osteoblasts thanks to α5β1 integrin and actin filaments.

The ability of fibronectin to link with α5β1 integrin has currently been considered as the most common pathway for the internalization of *S. aureus* in endothelial cells and osteoblasts (Figure 2). Though this mechanism is specific to *S. aureus* (Khalil et al., 2007), deleting FnBP A/B in *S. aureus* does not completely abrogate its internalization by osteoblasts, suggesting the involvement of other mechanisms in this process (Ahmed et al., 2001). Cna and Bbp actually favor the FnBP-mediated internalization (Testoni et al., 2011) whereas other MSCRAMMs of *S. aureus* are involved in integrin-mediated internalization (Hirschhausen et al., 2010; Zapotoczna et al., 2013). Other BEM components like collagen or bone sialoprotein, also bind to multiple integrins supporting the possibility of still unknown “MSCRAMM/BEM component/integrin” bridge systems to initiate the internalization of *S. aureus* by osteoblasts (Grzesik and Robey, 1994).

Independently of the extracellular events, the involvement of the cytoskeletal elements, particularly actin microfilaments, has been shown during the internalization of *S. aureus* (Ellington et al., 1999; Jevon et al., 1999; Figure 2). In addition, the internalization process of *S. aureus* can occur with dead bacteria but not dead osteoblasts. This suggests that the internalization process is more an active cellular mechanism rather than an active bacterial mechanism (Hudson et al., 1995).

Internalization of *S. aureus* by osteoblasts is a key element in the spreading of the infection. It allows *S. aureus* to persist inside osteoblasts—protected from the immune system—and it gives to *S. aureus* the opportunity to sustain the infection.

## Intracellular *S. aureus* and persistence into osteoblasts

*S. aureus* can be localized in vesicles once inside the osteoblasts (Figure 2). A co-localization of fluorescent intracellular *S. aureus* and a lysosomal-associated membrane marker highlighted the trafficking of live bacteria into the late endosomal/lysosomal vesicles in osteoblasts. This hints to suggest that *S. aureus* survives inside the vesicles (Jauregui et al., 2013). Moreover, a recent study shows that internalization of *S. aureus* is higher in macrophages—a typical professional phagocytic cells—than in osteoblasts whereas the percentage of intracellularly surviving *S. aureus* was higher in osteoblasts than in macrophages (Hamza and Li, 2014). As non-professional phagocytic cells, the lack of an effective intracellular bacterial clearance program in osteoblasts may allow *S. aureus* to survive longer inside the latter. The ability to survive inside osteoblasts is nevertheless explained by the exit of *S. aureus* from the vesicles and its release in the cytoplasm to escape proteolytic activity of the lysosome.

There are several types of membrane-damaging factors such as hemolysins, bi-component leukocidins and phenol soluble modulins (PSM) among *S. aureus* arsenal of virulence factors (reviewed by Vandenesch et al., 2012). These factors are involved in the escape of *S. aureus* from intracellular vesicles.

PSMα—a member of the PSM family—has been observed to favor the escape of *S. aureus* from vesicles to the cytoplasm in different types of cell line (Grosz et al., 2014). In Hela cells, a similar mechanism of vesicular escaping has been also identified for either PSMβ or δ-toxin. These two members of the PSM family make the escape possible from the vesicles in synergy with β-toxin (Giese et al., 2011).

PSMα has been shown to mediate the cytotoxicity for intracellular *S. aureus* in osteoblast/*S. aureus* specific interaction (Cassat et al., 2013; Rasigade et al., 2013). Valour et al. more recently demonstrated that lack of δ-toxin in clinical isolates from *S. aureus* bone and joint infections was correlated with the chronicity of the infection (Valour et al., 2015b).

A tight relationship exists between vesicular escape, cytoxicity, and membrane-damaging virulence factors. We can easily assume that PSMs allow the escape from the vesicle and then acts on the cell membrane so as jeopardize osteoblasts. This phenomenon needs some investigations with regards to the *S. aureus*/osteoblast interaction.

In addition to the vesicular escape, intracellular persistence of *S. aureus* inside osteoblasts is also supported by the presence of small colony variants (SCV). SCVs are a slow-growing subpopulation of bacteria with atypical colony morphology on agar plates and unusual biochemical characteristics. SCVs consist of higher capacities for intracellular persistence and are less liable to antibiotics than their wild-type counterparts (Proctor et al., 2006). Involvement of SCVs in intracellular persistence might be due to a better susceptibility to host uptake and/or a selection of SCVs due to intracellular environment properties (Proctor et al., 2014). SCVs were observed in *in vitro* models of osteoblast/*S. aureus* interaction (Valour et al., 2015a,b). A high percentage of SCVs was correlated with less cytoxicity of osteoblasts in isolates from chronic osteomyelitis with *S. aureus* (Kalinka et al., 2014). In this context, Sigma Factor SigB of *S. aureus* was shown to be an essential factor that leads to *S. aureus* to switch from the highly aggressive phenotype that settles an acute infection to a silent SCV-phenotype that leads to a long-term intracellular persistence (Tuchscherr et al., 2015). A continuous upregulation of SigB expression during intracellular persistence was correlated with the upregulation of adhesins and downregulation of toxins, which are characteristic for SCVs (Tuchscherr and Löffler, 2015). The impact of SigB in *S. aureus* internalization by osteoblasts was already observed more than a decade ago (Nair et al., 2003). Moreover, SCVs were observed to rapidly revert to the fully virulent wild-type form when leaving the intracellular location and infecting new cells (Tuchscherr et al., 2011).

Membrane-damaging virulence factors and SCVs are major parameters in intracellular persistence of *S. aureus* inside osteoblasts. On the one hand, PSMs make the escape from the intracellular vesicle to the cytoplasm easier but also increase the osteoblast death. On the other hand, SCVs can easily persist inside osteoblasts thanks to their lowered virulence factors.

## Receptors involved in the interaction between *S. aureus* and osteoblasts

In addition to binding to the BEM, *S. aureus* can also directly stimulate osteoblasts, mostly through its PAMPs, leading to various cellular responses (summarized in Table 2). The PAMPs can interact with the osteoblasts pattern recognition receptors (PRR) such as Toll-like receptors (TLR) and nucleotide-binding oligomerization domains (NOD) like receptors (NLR), as well as with the tumor necrosis factor receptor 1 (TNFR-1; Figure 2).

**Table 2:** ***In vitro* consequences of *Staphylococcus aureus*/osteoblast interaction**.

**Cells**	**Strains**	**MOI**	**Observations**	**Author/Year**
MNCOs, NHOs	UAMS-1	25:1, 75:1, 250:1	Upregulation of expression and release of IL-6 and IL-12 in infected osteoblasts	Bost et al., 1999
MNCOs, NHOs	UAMS-1	25:1, 75:1, 250:1	Upregulation of expression and release of GM-CSF and G-CSF in infected osteoblasts	Bost et al., 2000
MC3T3-E1	UAMS-1	250:1	Intracellular *Staphylococcus aureus* induces apoptosis of osteoblasts	Tucker et al., 2000
MNCOs, NHOs	UAMS-1	25:1, 75:1, 250:1	Induction of expression and release of TRAIL in infected osteoblasts	Alexander et al., 2001
MNCOs, NHOs	UAMS-1	25:1, 75:1, 250:1	Upregulation of expression and release of MCP-1 in infected osteoblasts, but not IL-3	Bost et al., 2001
MNCOs, NHOs	UAMS-1,	25:1, 75:1, 250:1	Increase of phosphorylation of ERK 1 and 2 and activation of c-jun in *Staphylococcus aureus*-infected osteoblasts	Ellington et al., 2001
MG-63, NHOs	Clinic	10:1, 1:1	Upregulation of expression and release of IL-8, IP-10, RANTES, MCP-1 in infected osteoblasts	Wright and Friedland, 2002
MNCOs, NHOs	UAMS-1	75:1, 250:1	Upregulation of expression and release of IP-10 in infected osteoblasts	Gasper et al., 2002
MNCOs, NHOs	UAMS-1	25:1, 75:1, 250:1	Upregulation of expression of IL-1β and IL-18 in infected osteoblasts, but no release of the related proteins in spite of the presence of caspase I	Marriott et al., 2002
MNCOs, NHOs	UAMS-1	25:1, 75:1, 250:1	Expression of TRAIL in infected osteoblasts induces apoptosis and activation of caspase-8	Alexander et al., 2003
MNCOs, NHOs	UAMS-1	25:1, 75:1, 250:1	Upregulation of expression and production of CD40 in infected osteoblasts and mediation of cytokine induction	Schrum et al., 2003b
MNCOs, NHOs	UAMS-1	25:1, 75:1, 250:1	Upregulation of mRNA and surface expression of MHC II in infected osteoblasts	Schrum et al., 2003a
MNCOs	Bacterial DNA	-	Induction of osteoclastogenesis by ligation of bacterial DNA upon TLR-9 in osteoblasts	Zou et al., 2003
MG-63, NHOs	Clinic	10:1, 1:1	Effect of Th2 cytokines, PGE2 and dexamethasone on release and expression of RANTES, MCP-1, IP-10, IL-8 from infected osteoblasts	Wright and Friedland, 2004
MNCOs	UAMS-1	25:1, 75:1, 250:1	Stimulation of Nod1 and Nod2 by intracellular *Staphylococcus aureus* and related cytokine release	Marriott et al., 2005
MNCOs, NHOs	UAMS-1	125:1	Decrease of sensibility to antibiotics for intracellular established *Staphylococcus aureus*	Ellington et al., 2006
MNCOs, NHOs	UAMS-1, Lab	25:1, 75:1	Expression of TRAIL is observed in both infected and uninfected osteoblasts in infected cultures	Reott et al., 2008
MNCOs,	UAMS-1, Clinic	25:1, 75:1, 250:1	Upregulation of expression and production of RANK-L and production of PGE2 via COX-2 activation in infected osteoblasts	Somayaji et al., 2008
hFOBs	-	Supernatant	Up-regulation of the production of HBD-2 by osteoblasts challenged with *Staphylococcus aureus* supernatants	Varoga et al., 2008
SaoS-2, NHOs	-	Supernatant	Production of HBD-3 by osteoblasts via TLR2 after stimulation with *Staphylococcus aureus* supernantant	Varoga et al., 2009
MNCOs	UAMS-1	25:1, 75:1, 250:1	Modulation of osteoblast response to *Staphylococcus aureus* through NOD2	Chauhan and Marriott, 2010
SV40 hOBs	UAMS-1	250:1	Release of MCP-1 and IL-6 in infected osteoblasts through NF-kB regulation	Ning et al., 2010
SV40 hOBs	UAMS-1	250:1	Necessity of attachment of *Staphylococcus aureus* to osteoblasts but not internalization for activation of NF-kB	Ning et al., 2010
MNCOs, NHOs	UAMS-1, Lab	25:1, 75:1, 250:1	Expression of TRAIL death receptors, DR4 and DR5, and decrease of OPG production in infected osteoblasts	Young et al., 2011
MC3T3-E1	Lab	-	Induction of osteoblast apoptosis via Caspase-3 and expression of RANK-L thanks to the binding of SpA from *Staphylococcus aureus* to TNFR-1 of osteoblasts	Claro et al., 2011
MC3T3-E1	Lab	-	Inhibition of the osteoblast proliferation, induction of the migration of pre-osteoclasts and osteoclastogenesis through SpA from *Staphylococcus aureus*	Widaa et al., 2012
MC3T3-E1, SaoS-2	Clinic	Supernatant	Dose-dependent cytotoxicity of PSMα of *Staphylococcus aureus* on osteoblasts	Cassat et al., 2013
MC3T3-E1	Lab	-	SpA binds to TNFR-1 which activates NF-kB and the release of IL-6	Claro et al., 2013
MC3T3-E1	Lab	100:1	PVL, SpA and Coa of *Staphylococcus aureus* induce apotosis and upregulate RANK-L expression in osteoblasts	Jin et al., 2013
MG-63	Lab, clinic	100:1	Killing effect of PSMα secreted by intracellular *Staphylococcus aureus* on osteoblasts	Rasigade et al., 2013
MC3T3-E1	Lab	Supernatant	Induction of production of MDB-14 (a murine ortholog HDB-3) by *Staphylococcus aureus*-infected osteoblasts through MAPK and NF-kB pathways	Zhu et al., 2013
NHOs	UAMS-1, clinic	Biofilm supernatant	Decrease of osteoblast viability, inhibition of osteogenic differentiation and increase of RANKL expression by osteoblasts by biofilm components of *Staphylococcus aureus*	Sanchez et al., 2013
MC3T3-E1	Lab	100:1	Apoptosis and decrease of osteogenic differentiation in response to *Staphylococcus aureus* through TLR2 expression and JNK activation	Chen et al., 2014
Primary osteoblasts	Lab	20:1, 100:1, 500:1	Production of CCL2 and CXCL3 by osteoblasts following stimulation by *Staphylococcus aureus*	Dapunt et al., 2014
MC3T3-E1	Lab	100:1	Expression of miR-24 was significantly down-regulated in *Staphylococcus aureus*-infected osteoblasts compared with untreated control cells. Overexpression of miR-24 could reduce the effects of *Staphylococcus aureus*	Jin et al., 2015

MOI, Multiplicity of infection (number of bacteria per cell); MNCOs, Mouse neonate calvarial osteoblasts; NHOs, Normal human osteoblasts; MC3T3-E1, Mouse calvaria cell line; MG-63, Human osteosarcoma cell line; SaoS-2, Human osteosarcoma cell line; hFOBs, Human fetal osteoblastic cell line; SV40 hOBs, Human osteoblastic cell line; Lab, Laboratory strains of Staphylococcus aureus (purchased from ATCC, NCTC,… ); Clinic, Strain of Staphylococcus aureus isolated from osteomyelitic patients; UAMS-1, Most studied laboratory strain of Staphylococcus aureus in bone infection models.

TLR is a family of thirteen membrane-bound PRRs that can interact with different bacterial components (Kawai and Akira, 2010). TLR-2, TLR-4, TLR-5, and TLR-9 have been observed in osteoblasts. TLR-2 is an extracellular receptor that can recognize the PAMPs from *S. aureus* (Fournier, 2012). It induces the release of AMPs as well as the induction of osteoblast apoptosis following its activation by *S. aureus* (Varoga et al., 2009; Chen et al., 2014). TLR-4 and TLR-5 take part in responses toward Gram negative bacteria, by respectively recognizing LPS and flagellin but are not involved in *S. aureus*/osteoblast interaction (Kikuchi et al., 2001; Madrazo et al., 2003).

As opposed to TLRs −2, −4, and −5, TLR-9 is an intracellular member of the TLR family. It is bound to the membrane of intracellular vesicle. TLR-9 recognizes the CpG oligodesoxynucleotides of bacterial DNA and is able to detect the presence of *S. aureus* DNA (Zou et al., 2003). This can however occur but after the disruption or disintegration of *S. aureus* within the intracellular vesicles, leading to the release of its DNA that then binds to TLR-9 and induces the activation of signaling cascades (Wolf et al., 2011; Figure 2).

As above mentioned, intracellular *S. aureus* can also escape from a vesicle and be released into the cytosol of osteoblasts following its internalization. *S. aureus* can induce in this case a response through the NLRs, a family of 22 intracellular PRRs (Figure 2), NOD1, NOD2, NLRP3, and NLRC4 have been studied in osteoblasts among the latter family. Over-expression of NOD1 and NOD2 was assessed in osteoblasts infected by intracellular *S. aureus* and led intracellular signaling to be activated and to the production of immune molecules (Marriott et al., 2005). Another study focusing on NOD2 backs the idea that this NLR may serve differential roles in osteoblasts, promoting inflammatory responses to invasive bacteria while tempering cell responses to extracellular and/or passively internalized bacterial species, such as *S. aureus* (Chauhan and Marriott, 2010). Two other NLRs, NLRP3, and NLRC4, were also evidenced in osteoblasts. NLRP3 is expressed in osteoblasts whereas NLRC4 expression was not found. The role of NLRP3 in bacteria-related osteoblast death was studied for the invasive bacteria *Salmonella enterica* but not for *S. aureus* (McCall et al., 2008).

It has been recently reported that *S. aureus* can also interact through the extracellular TNFR-1 on epithelial cells and osteoblasts independently from PRRs such as TLRs and NLRs (Gómez et al., 2004; Claro et al., 2013). Originally described as an exclusive receptor for the Tumor Necrosis Factor α (TNF-α), it was shown that TNFR-1 interacts with the protein A of *S. aureus* and may cause the release of cytokines, the apoptosis of osteoblasts, or an unbalanced bone homeostasis (Claro et al., 2011, 2013; Widaa et al., 2012).

It was proved that many signaling pathways are activated in *S. aureus*-infected osteoblasts including ERK 1/2, JNK, c-Jun, AP-1, Rip2 Kinase and NF-κB following the interaction with PRRs or TNFR-1, (Ellington et al., 2001; Wright and Friedland, 2002; Marriott et al., 2005; Ning et al., 2010; Claro et al., 2013; Figure 2). *S. aureus* can virtually activate different signaling pathways through either extracellular or intracellular receptors. This combination of signaling mechanisms highlights the diversity and the complexity of the osteoblast response to various signals and/or bacterial infections.

## Implication of the *S. aureus-challenged* osteoblasts in the immune response

**Figure 3:**
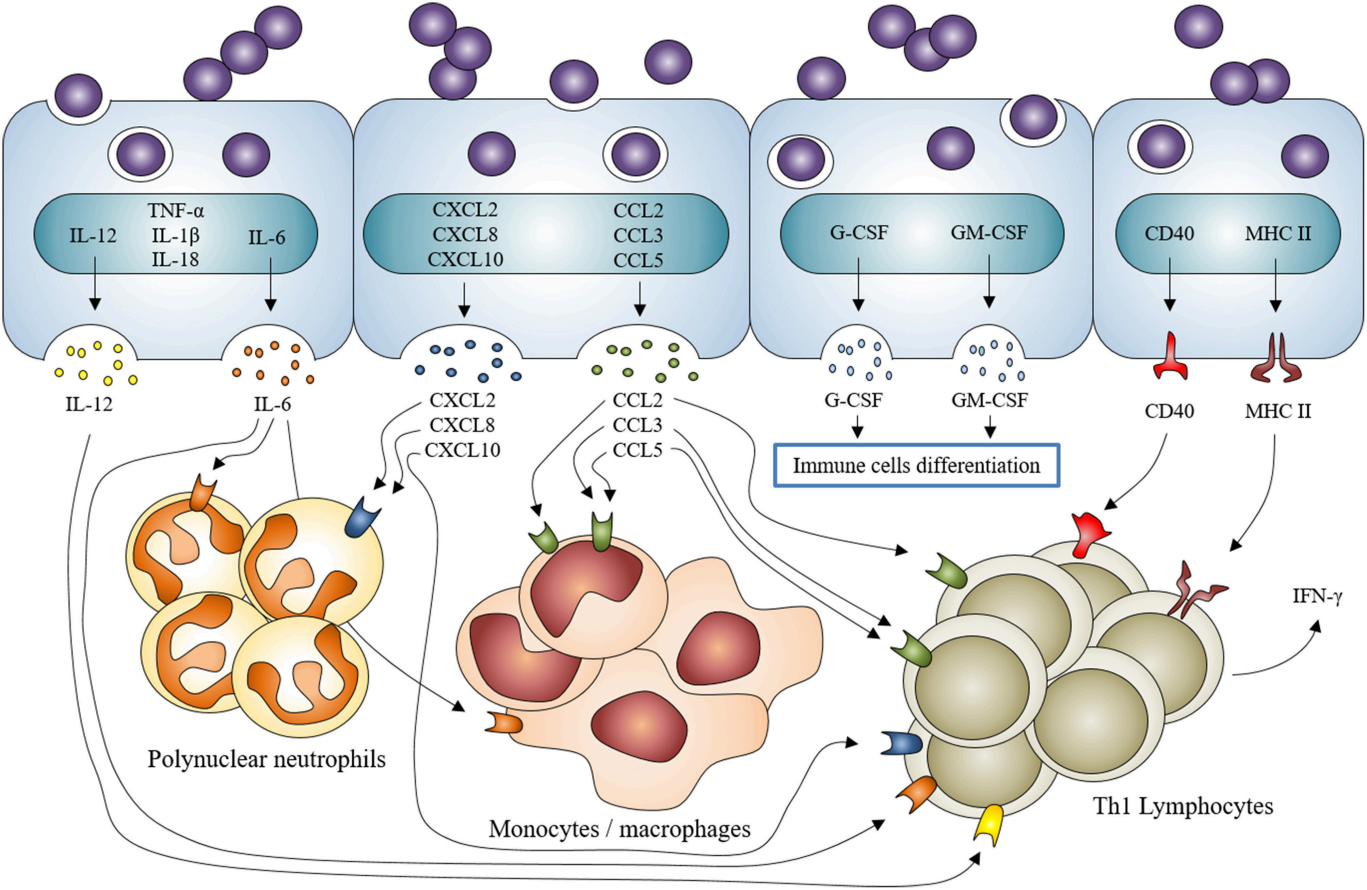
***Staphylococcus aureus* induction of inflammatory mediator production by osteoblasts and participation in the recruitment/activation of innate and adaptive immune cells**. *Staphylococcus aureus* interaction with osteoblasts increases the expression of cytokines IL-1β, IL-18, TNF-α, the production and release of IL-6, IL-12 and the expression and release of chemokines CXCL2, CXCL8, CXCL10, CCL2, CCL3, CCL5 and growth factors G-CSF and GM-CSF. It also increases the expression and production of CD40 and MHC II. All of this participates to the recruitment and activation of innate (neutrophils, monocytes/macrophages) and adaptive (lymphocytes) immune cells.

Following contact/internalization of *S. aureus*, the first line of defense of the osteoblasts is to secrete inflammatory factors like cytokines, chemokines and growth factors, all of which are able to activate and recruit immune cells from the innate or adaptive immune systems (Turner et al., 2014; Figure 3).

More specifically, the osteoblast production of IL-6, CCL2 (MCP-1), CCL3 (MIP-1α), and CCL5 (RANTES) increases following *S. aureus* infection. These mediators are able to recruit and activate macrophages (Bost et al., 1999, 2001; Ning et al., 2011; Dapunt et al., 2014). An over-production of G-CSF and GM-CSF—two growth factors that favor the maturation of leukocytes—has also been demonstrated (Bost et al., 2000). CXCL8 (IL-8) and CXCL2 (MIP-2α also called GRO-β)—two neutrophil chemoattractants - are also up-regulated during osteoblasts/*S. aureus* interaction (Wright and Friedland, 2002, 2004; Dapunt et al., 2014). Through releasing these mediators, infected osteoblasts participate in the innate immune response in an attempt to clear the bacteria.

Osteoblasts are furthermore deemed to take part in the adaptive response to *S. aureus*. Infected osteoblasts up-regulate their release of IL-12 and CXCL10 (IP-10)—two molecules that recruit and activate Th1 lymphocyte—(Bost et al., 1999; Gasper et al., 2002). *S. aureus*-challenged osteoblasts are also able to express CD40 and MHC II, both implicated in the initiation of Th1 lymphocyte response (Schrum et al., 2003a,b). T lymphocytes secrete IFN-γ—a hallmark of their activation—while interacting with *S. aureus*-infected osteoblasts (Bost et al., 1999). There are no reports that confirm or exclude a role of other adaptive immune cells such as Th17 lymphocytes or dendritic cells in the response of osteoblasts to *S. aureus*. This needs to be further investigated to figure out whether if osteoblasts have a real role in adaptive immune responses in osteomyelitis or not.

An over-expression of TNF-α, IL-1β, and IL-18 mRNAs has been proved but the related-protein release has not been observed yet (Littlewood-Evans et al., 1997; Marriott et al., 2002), thus we cannot assess the impact of *S. aureus*-infected osteoblasts on inflammatory processes through these mediators. These findings once combined suggest that osteoblasts infected by *S. aureus* may participate in the innate immune response as well as in the adaptive immune response likely through the initiation of a Th1 lymphocyte response. This is sustained by current studies focusing on the mutual interplay between immune and bone systems designated as “osteoimmunology” (reviewed in Takayanagi, 2007, 2015).

*In vitro* experiments were nonetheless mostly performed using osteoblastic cell lines or marketed normal osteoblasts. This may lead to an overstatement of the results, as such models diverge from the *in vivo* pathophysiological scenario. As described in Table 2, most of these results were obtained from different *in vitro* studies, with various conditions of cell culture or multiplicity of infection. The high multiplicities of infection or long time periods are globally needed for a release of cytokines/chemokines by osteoblasts. An increase of mRNA expression happens 6 or 8 h after the interaction of *S. aureus* with osteoblasts whereas the relative protein release starts from 24 to 48 h after the interaction. Those results justify the idea that inflammatory response by osteoblasts should not be pivotal in osteomyelitis caused by *S. aureus*. In addition, *in vivo* animal models of osteomyelitis showed that professional immune cells are rapidly recruited after infection (Yoshii et al., 2002; Horst et al., 2012; Niska et al., 2012). Quantifying the release of mediators especially produced by osteoblasts but not from recruited immune cells appears to be a hard thing to do. These recruited immune cells might likewise be more able to release large amounts of IL-1β, IL-6 and, TNF-α than osteoblasts. Thinking this through, we propose the osteal macrophages—also called OsteoMacs—to act as the initiator of inflammation in osteomyelitis. Those bone resident macrophages—which are different from osteoclast precursors—are localized into osteal tissue and play a role in both the bone formation and bone reparation and bone-related diseases as previously observed (Chang et al., 2008; Pettit et al., 2008). OsteoMacs seem to be more likely to initiate an inflammatory response in osteomyelitis than osteoblasts since they are professional resident immune cells.

## *S. aureus* impact on osteoblast activity and viability

*S. aureus* can also induce bone loss as well as a production of inflammatory mediators from osteoblasts. This phenomenon is partially due to a decrease of osteoblast activity and to their death following bacterial insult. *S. aureus* is known to inhibit osteoblast activity and differentiation. Several studies have shown a decrease of proliferation, a decreased alkaline phosphatase activity, and a lower expression of BEM components such as type I collagen, osteocalcin, osteopontin, and osteonectin in *in vitro* models of *S. aureus*/osteoblast interaction (Claro et al., 2011; Widaa et al., 2012; Jin et al., 2013). *S. aureus* is also known to prevent the mineralization process, observed through a lower calcium deposition in infected osteoblast cultures compared to their non-infected counterparts (Claro et al., 2011; Jin et al., 2013; Sanchez et al., 2013; Chen et al., 2014).

Controversies arose from Somayaji et al. work in which they have figured out that UV-killed *S. aureus* coated on titanium alloys favored osteoblast adhesion, osteoblast differentiation and mineralization (Somayaji et al., 2010). These results in opposition with all the previous statements suggest that the effect of *S. aureus* on osteoblast activity is not totally elucidated. Jin et al. recently highlighted that effects of *S. aureus* on osteoblast activity could be dependent of microRNAs (Jin et al., 2015). Relationship between osteoblast response to *S. aureus* and the microRNAs production could be a new interesting approach to understand osteomyelitis at a cellular level and decipher which components are able to whether decrease or increase osteoblastic activity.

**Figure 4:**
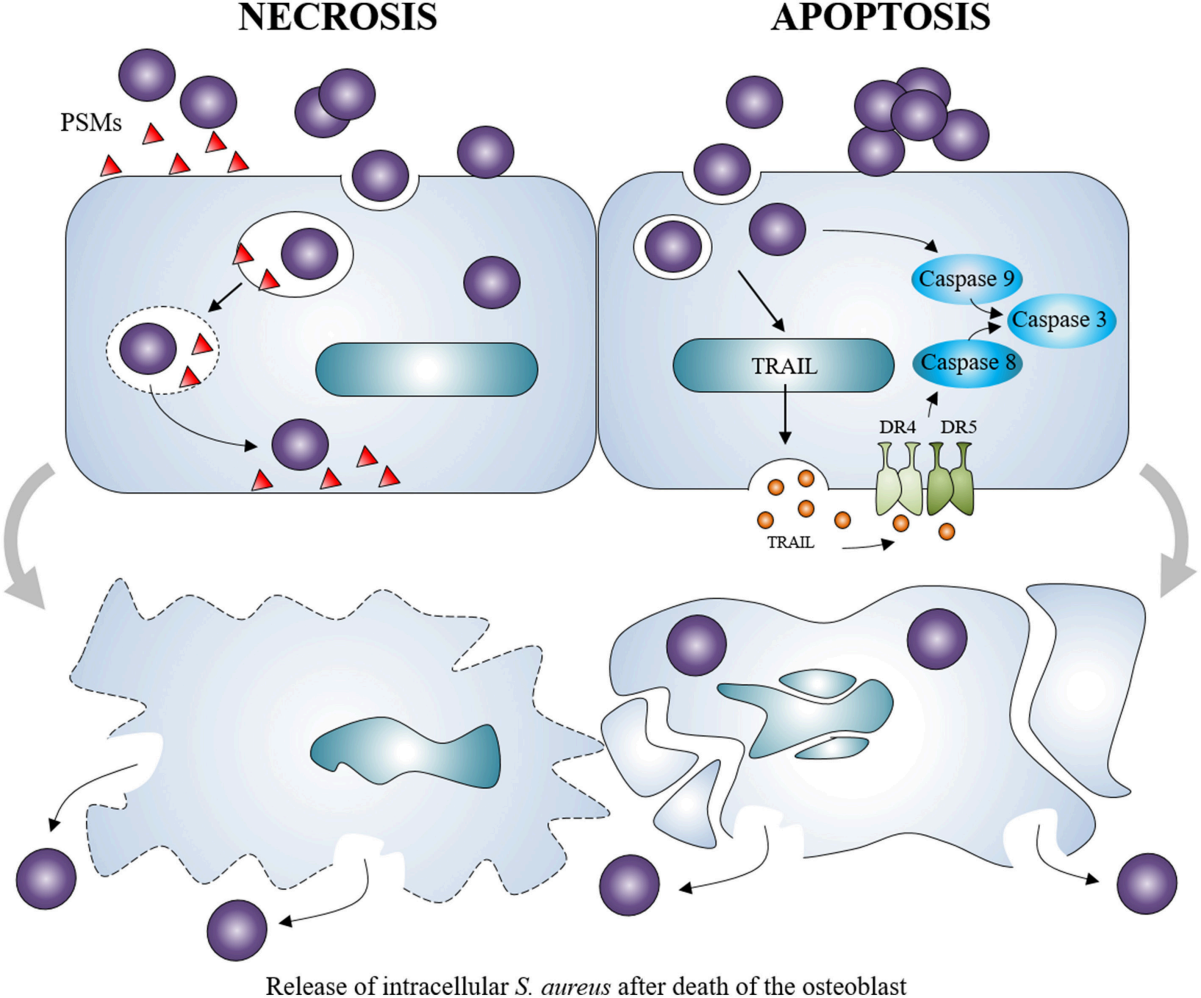
***Staphylococcus aureus* induction of osteoblast death**. *Staphylococcus aureus* can cause the necrosis of osteoblasts through the release of membrane-damaging virulence factors such as PSMs. It can also induce the apoptosis of osteoblasts through intrinsic and extrinsic caspase pathways. Both can lead to the release of intracellular *Staphylococcus aureus*, which can re-infect other osteoblasts.

*S. aureus* can also cause apoptosis of osteoblasts regardless to specific osteoblastic activity. This is observed following *S. aureus* infection of various cell types including endothelial cells, epithelial cells or osteoblasts (Menzies and Kourteva, 1998; Kahl et al., 2000; Tucker et al., 2000). In *S. aureus*-infected osteoblasts, apoptosis appeared to be dependent on the release of the tumor necrosis factor-related apoptosis-inducing ligand (TRAIL; Alexander et al., 2001). TRAIL interacts with death receptors DR4 and DR5 expressed in infected osteoblasts (Young et al., 2011), leading to the induction of apoptotic pathways. In the situation of *S. aureus*-infected osteoblasts, an activation of the apoptosis intrinsic pathway through caspase-9 (Jin et al., 2013) and the extrinsic pathway through caspase-8 was observed (Alexander et al., 2003), both of them leading to activation of caspase-3 and apoptosis (Claro et al., 2011; Figure 4).

Osteoblast death was also associated with above-cited toxins produced by *S. aureus* like PSMα, PSMβ, and δ-toxin (Cassat et al., 2013; Rasigade et al., 2013; Valour et al., 2015b). These virulence factors have a membrane-damaging activity which suggests that *S. aureus*-induced osteoblast death can also be independent of apoptosis. Non-apoptotic cell death has been observed in presence of α-toxin—another membrane-damaging virulence factor of *S. aureus*—in an *in vitro* cell line model (Essmann et al., 2003).

Death induction in osteoblasts is an important feature of *S. aureus*-related osteomyelitis. On the one hand, it leads to lowered bone formation activity and on the other hand, the death of osteoblasts may allow the release of the intracellular viable *S. aureus*, which are able to re-infect surviving osteoblasts and exacerbate the infection (Ellington et al., 2003).

## *S. aureus*/osteoblasts interaction and osteoclastogenesis

Bone loss in osteomyelitis is also caused by an increased formation and activation of osteoclasts in addition to the death of osteoblasts. A tight balance between bone formation by osteoblasts and bone resorption by osteoclasts is essential for the strength and integrity of bones. We have so far reviewed a role of *S. aureus* in decreasing osteoblast activity and inducing osteoblast death, hinting an impaired osteoblast/osteoclast balance necessary for proper bone formation.

**Figure 5:**
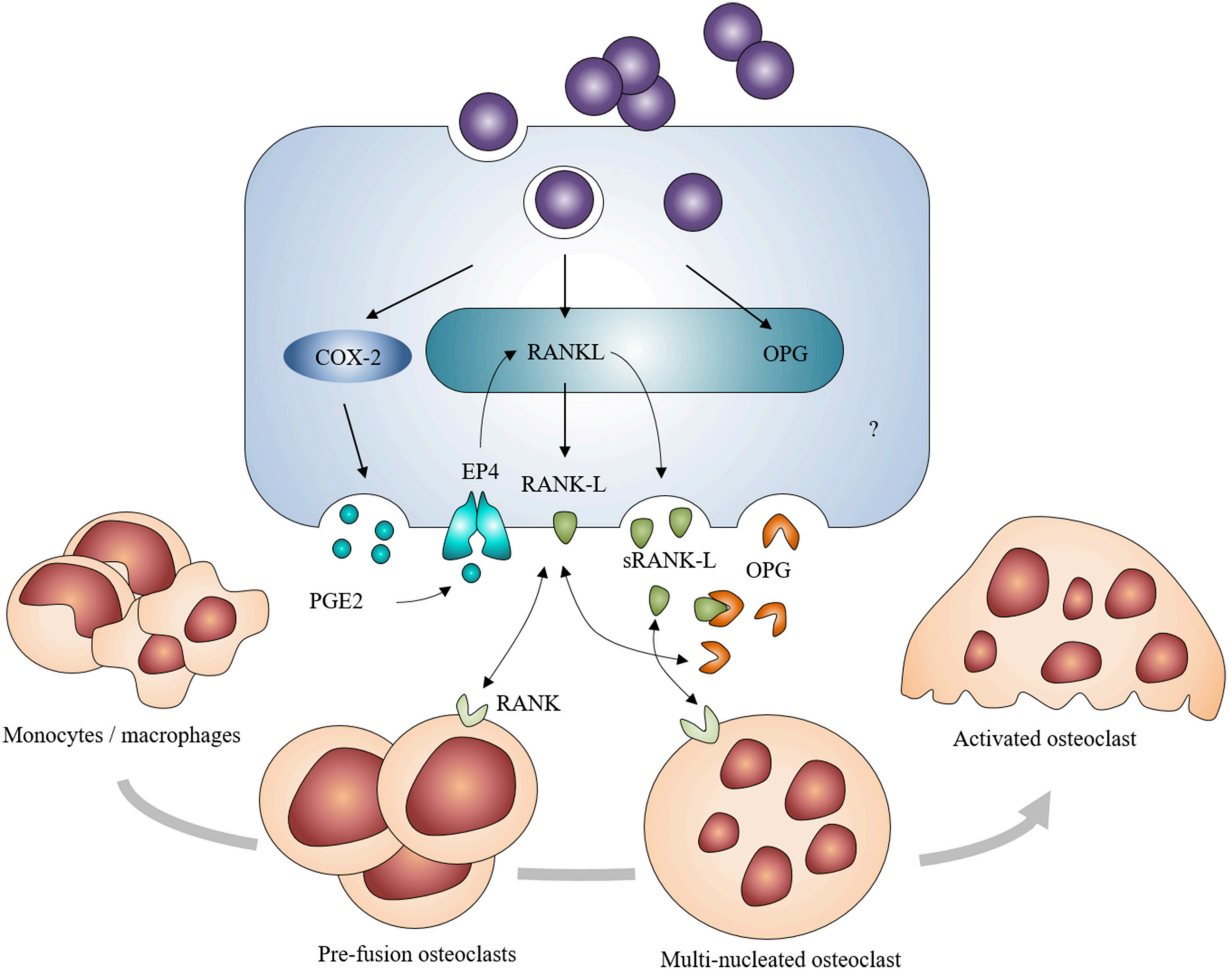
**Induction of osteoclastogenesis by *Staphylococcus aureus*-challenged osteoblasts**. *S. aureus*-challenged osteoblasts increase their expression and production of RANK-L, directly or through the COX-2/PGE2 pathway. It leads to an excessive formation and activation of osteoclasts and to a severe bone resorption in addition to a decreased production of OPG.

Osteoblasts are producers of receptor activator of NF-κ B ligand (RANK-L) and osteoprotegerin (OPG). RANK-L interacts with its receptor RANK on monocyte/macrophage precursors to drive their differentiation toward osteoclasts. OPG is a soluble decoy receptor that targets RANK-L to limit osteoclastogenesis (Boyle et al., 2003). In the case of *S. aureus* infection, osteoblasts increase their production of membrane-bound RANK-L and sRANK-L—a soluble and minor form of RANK-L—whereas OPG production by osteoblasts decreases following *S. aureus* infection despite an absence of modulation of the OPG mRNA synthesis (Somayaji et al., 2008; Claro et al., 2011; Young et al., 2011; Widaa et al., 2012; Figure 5). It is therefore thought that *S. aureus* is able to indirectly increase osteoclastogenesis as a result of its interaction with osteoblasts. An increase of prostaglandin E2 (PGE2) production has furthermore been demonstrated in *S. aureus*-infected osteoblasts (Somayaji et al., 2008). PGE2—a hormone-like molecule and enzymatic product of the cyclo-oxygenase 2 (Cox-2)—acts in an autocrine and paracrine manner to upregulate the production of RANK-L through activation of the EP4 receptor in osteoblasts (Figure 5). PGE2 up-regulated by *S. aureus* thus acts as a complementary stimulating effect on osteoblasts to release osteoclastogenic factors (Johansen et al., 2012). Recent studies nevertheless showed that *S. aureus* or its components can also favor bone resorption through interaction with osteoclast cell lineage. *S. aureus* provoked the enhancement of the osteoclast differentiation and the release of pro-inflammatory cytokines (Kim et al., 2013). These cytokines enhanced the bone resorption capacity of uninfected mature osteoclasts and promoted osteoclastogenesis of the uninfected precursors (Trouillet-Assant et al., 2015). Further studies are yet needed to fully fathom the interactions between *S. aureus* and osteoclasts and the mechanisms involved in the latter interactions.

## Osteoblast self-defense against *S. aureus*

Infected osteoblasts can release AMPs in order to defend themselves against *S. aureus*. These molecules, mostly cationic, are secreted by various types of cells and are able to neutralize bacteria. They are classified in three categories: α-defensins, β-defensins and cathelicidin-related molecules—with LL-37 as the only member in humans—(reviewed in Mansour et al., 2014). It was shown that human osteoblasts increase their release of human β-defensin (HBD) 2 and HBD-3 following stimulation by *S. aureus* culture supernatants, (Varoga et al., 2008, 2009). The release of MBD-14—the murine ortholog of HBD-3—was also seen in infected murine osteoblasts (Zhu et al., 2013). A higher expression of HBD-3 and MBD-14 was also found in human interfacial membrane surrounding implants infected by *S. aureus* and in a murine model of osteomyelitis, respectively (Zhu et al., 2014). LL-37 is moreover secreted by the human bone marrow mesenchymal stem cells (Krasnodembskaya et al., 2010) and described to exhibit a killing effect on *S. aureus* inside osteoblasts (Noore et al., 2013). The use of exogenous LL-37 in osteomyelitis is however uncertain as it might have deleterious effects on osteoblasts (Säll et al., 2013; Svensson et al., 2015). As for α-defensins, their production is mostly attributed to neutrophils or Paneth cells, and not to osteoblasts or other stromal cells (Mansour et al., 2014). The use of exogenous AMPs could be a promising and effective way to treat osteomyelitis. Synthetic compounds similar to AMPs were produced and relevant as treatments toward bacterial infection, and notably in a *S. aureus* infection model (Nijnik et al., 2010). They can be added by the way to supports like titanium alloys, thus enlarging their possible use to fight against implant-associated osteomyelitis (Kazemzadeh-Narbat et al., 2013). Up to now, the emergence of resistance to AMPs was considered low, virtually because of the complexity of their mode of action (Jenssen et al., 2006). A recent review however highlights that *S. aureus* holds mechanisms of resistance to AMPs (Joo and Otto, 2015). As a matter of fact, we deem this moderates the brilliant future for AMPs in anti-staphylococcal treatments be it in bone or elsewhere.

## Antibiotic chemotherapies at *S. aureus*/osteoblasts specific scale

Clinical guidelines for the treatment of osteomyelitis with *S. aureus* generally recommend the use of a combination of antibiotics consisting of penicillin M—i.e., oxacillin—combined with an aminoglycoside—i.e., gentamicin—or to rifampicin. The uses of glycopeptide—i.e., vancomycin—combined with linezolid or rifampicin, of clindamycin combined with rifampicin, or of fluoroquinolone—i.e., ofloxacin—combined with rifampicin are considerable options for the treatment of osteomyelitis with meticillin-resistant *S. aureus* (MRSA; Lew and Waldvogel, 2004; Hatzenbuehler and Pulling, 2011; Osmon et al., 2013; Lima et al., 2014).

The internalization of *S. aureus* inside osteoblasts however represents a major challenge in the treatment of osteomyelitis. The ability of the recommended antibiotics to kill intracellular *S. aureus* is the cornerstone for relevant treatment of osteomyelitis. In this context, studies have assessed the effects of these antibiotics in *in vitro* models of infection. A treatment with gentamicin, vancomycin or daptomycin decrease the number of intra-osteoblastic bacteria, without totally eradicating them, even at high concentrations. The rifampicin used in clinical concentrations has on the contrary resulted to eradicate intracellular *S. aureus* inside cultured osteoblasts (Mohamed et al., 2014; Valour et al., 2015a). The treatment has to start but very early since a 12 h-delayed rifampicin treatment of *S. aureus*-challenged osteoblasts significantly decreased its effectiveness in killing intracellular bacteria by 100-fold (Ellington et al., 2006). In this case, the surface of *S. aureus* is altered after exposure to the intracellular osteoblast environment as Ellington et al. described it. A thick capsular material appears on intra-osteoblastic *S. aureus* that could explain the resistance to rifampicin. Specific mechanisms that describe the alteration of the surface of *S. aureus* by osteoblast intracellular environment have not been determined yet but we can suggest that SCVs may be involved in this phenomenon. One can say that further investigations need to be carried out. Combined therapy using rifampicin and ultrasound has been recently proposed to enhance the intracellular killing relevancy but it seemed that ultrasounds had a negative impact on the viability of cultured osteoblasts (Shi et al., 2013). This process is even more difficult to transpose to clinical applications.

Recent studies investigated the effect of tigecyclin, teicoplanin, ofloxacin, and linezolid. These antibiotics—which are recommended in the treatment of osteomyelitis—have an intermediate intracellular antimicrobial effectiveness, higher than gentamicin or vancomycin and lower or equivalent to rifampicin (Kreis et al., 2013; Valour et al., 2015a). Ofloxacin, daptomycin, and vancomycin were also deemed to limit intracellular SCVs emergence (Valour et al., 2015a). The concentration however used in antibiotic treatment is also a critical point. Sub-inhibitory concentrations of oxacillin, moxifloxacin, and linozelid increase the fibronectin-mediated binding of *S. aureus* whereas sub-inhibitory concentrations of rifampicin still have decreasing effects on its binding (Rasigade et al., 2011). Radezolid and naficillin-loaded nanoparticles have also been investigated for their ability to kill *S. aureus* inside cultured osteoblasts (Pillai et al., 2008; Lemaire et al., 2010). Radezolid has shown a higher bactericidal activity compared to linezolid and an effective killing of intracellular bacteria was observed with naficillin-loaded nanoparticles.

All these recent studies shed light on the importance of intra-osteoblastic penetrance of antibiotics. This property is essential for the development of new therapeutics against osteomyelitis caused by *S. aureus*.

## Conclusion

**Figure 6:**
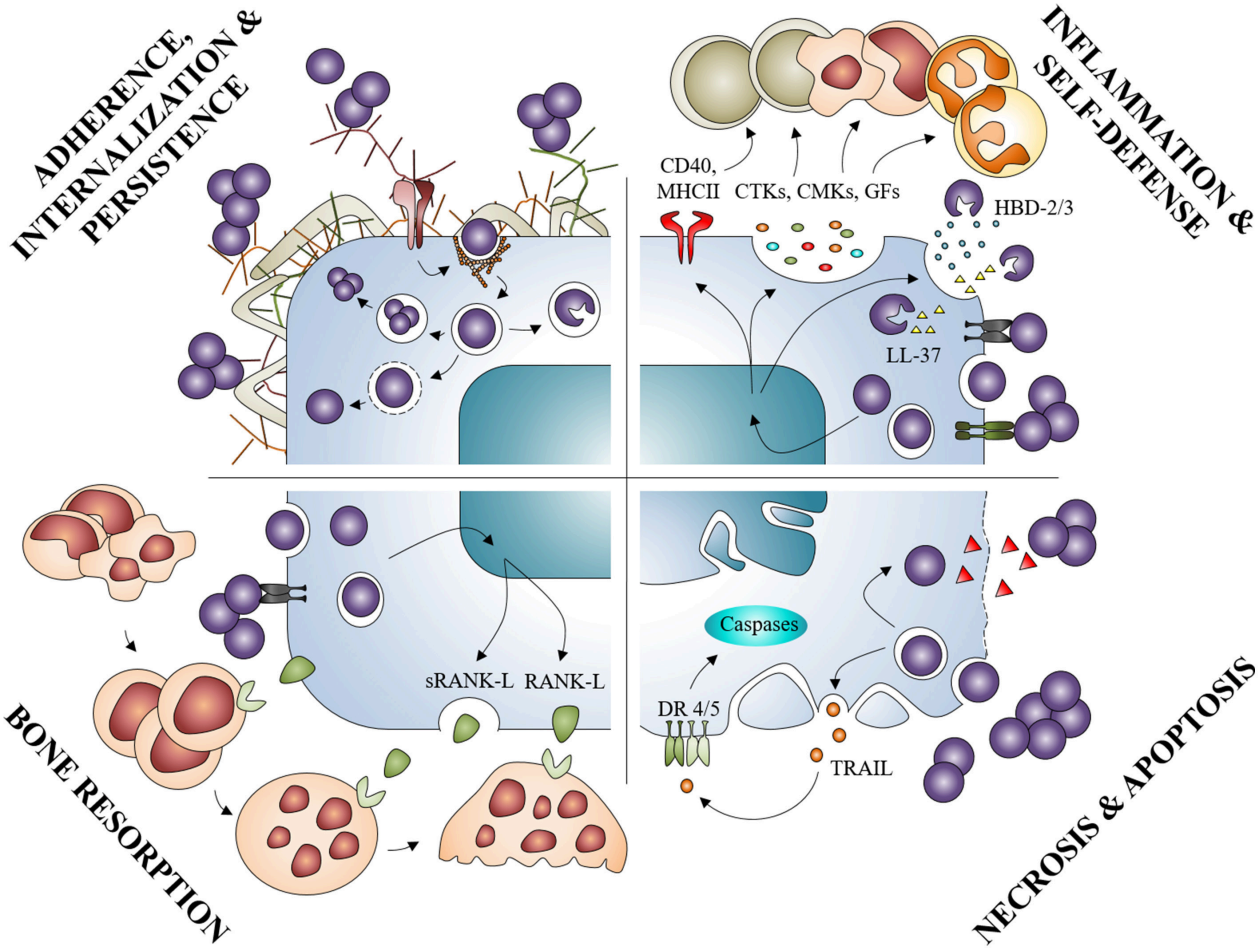
**Summary diagram of the osteoblasts responses in presence of *Staphylococcus aureus***. *S. aureus* can interact with osteoblasts and provoke: inflammation by increased release of mediators such as cytokines (CTKs), chemokines (CMKs), or growth factors (GFs); osteoblast self-defense by the production of AMPs (HBD-2/3, LL-37); osteoblasts death through apoptosis or necrosis; bone resorption by activation of the RANK/RANK-L complex.

Osteoblast infection by *S. aureus* can be defined as a 4-step process from mostly *in vitro* models. First, *S. aureus* binds to the BEM and to osteoblasts. Through attachment, *S. aureus* can be internalized and survive within the cell switching notably to a SCV phenotype. Then, *S. aureus* or its components may potentially modulate the production of cytokines, chemokines or AMPs, through binding to extracellular and/or intracellular receptors. Infection with *S. aureus* eventually induces inflammatory cell recruitment, which, combined with increased osteoclastogenesis and osteoblast death, leads to a massive bone loss (Figure 6).

Nevertheless, the defined role of infected osteoblasts in inflammatory response results from *in vitro* experiments that were mostly done with osteoblastic cell lines or marketed normal osteoblasts (Table 2) and diverges from the *in vivo* pathophysiological scenario. Cytokines/chemokines released by infected osteoblasts must only be a drop in the bucket compared to those released by neutrophils and macrophages/monocytes during osteomyelitis caused by *S. aureus*. From this point of view, osteoblasts thought to take part in global inflammatory response but without having a decisive role.

On the other side, internalization and intracellular persistence appears to us as the key stages of *S. aureus*/osteoblast interaction. Escape from intra-osteoblastic vesicle, switching to a SCV phenotype and osteoblast death following membrane damages are important phenomena to understand the evolution from acute to chronic osteomyelitis.

The *S. aureus* infection of the osteoblast is in the end a very complex phenomenon with a myriad of actors (Figure 6). To focus on one specific step allows us to better understand a precise mechanism but does not reflect the complexity of the entire infectious process, in the same way that focusing only on osteoblasts and avoiding other cells from bone tissues limits and biases our understanding. The use of new research models is necessary to complete the information obtained from the previous osteoblast *in vitro* infection models.

It is clear to us that more investigations using patient isolated *S. aureus*/primary osteoblasts *in vitro* models are needed and that systemic *in vivo* models close to the human scenarios of infection need to be optimized to consider new therapeutic approaches for this disease of high morbidity. New treatments or preventive approaches need also to be developed so as to avoid the internalization of *S. aureus* inside osteoblasts, contain the local inflammatory process, and increase the osteoblasts self-defense.

## Author contributions

JJ compiled the publications and wrote the first drafts of the review under the supervision of his advisors FV and SG. All the authors contributed equally to the final submitted version.

## Funding

JJ is the recipient of a fellowship from the French Ministry for Higher Education and Research—*Ministère de l'Enseignement Supérieur et de la Recherche*.

### Conflict of interest statement

The authors declare that the research was conducted in the absence of any commercial or financial relationships that could be construed as a potential conflict of interest.

## Abbreviations

*S. aureus*: *Staphylococcus aureus*
BEM: bone extracellular matrix
AMP: antimicrobial peptide
MSCRAMM: microbial surface components recognizing adhesive matrix molecule
PAMP: pathogen-associated molecular pattern
PRR: pattern recognition receptor
NOD: nucleotide-binding oligomerization domain
NLR: NOD like receptor
TLR: toll like receptor
TNFR-1: tumor necrosis factor receptor 1
TNF-α: tumor necrosis factor α
NF-κB: nuclear factor kappa B
MHC: major histocompatibility complex
RANK-L: receptor activator of NF-κB ligand
OPG: osteoprotegerin
TRAIL: tumor necrosis factor-related apoptosis-inducing ligand
DR: death receptor
PGE2: prostaglandin E2
HBD: human β-defensin
MBD: mouse β-defensin.
